# Cistrome Explorer: an interactive visual analysis tool for large-scale epigenomic data

**DOI:** 10.1093/bioinformatics/btad018

**Published:** 2023-01-23

**Authors:** Sehi L’Yi, Mark S Keller, Ariaki Dandawate, Len Taing, Chen-Hao Chen, Myles Brown, Clifford A Meyer, Nils Gehlenborg

**Affiliations:** Department of Biomedical Informatics, Harvard Medical School; Department of Biomedical Informatics, Harvard Medical School; Department of Data Science, Harvard T.H. Chan School of Public Health, Dana-Farber Cancer Institute; Department of Data Science, Harvard T.H. Chan School of Public Health, Dana-Farber Cancer Institute; Center for Functional Cancer Epigenetics, Dana-Farber Cancer Institute; Department of Data Science, Harvard T.H. Chan School of Public Health, Dana-Farber Cancer Institute; Center for Functional Cancer Epigenetics, Dana-Farber Cancer Institute; Department of Medical Oncology, Harvard Medical School, Dana-Farber Cancer Institute, Boston, MA 02215, USA; Department of Data Science, Harvard T.H. Chan School of Public Health, Dana-Farber Cancer Institute; Department of Biomedical Informatics, Harvard Medical School

## Abstract

**Summary:**

The regulation of genes by cis-regulatory elements (CREs) is complex and differs between cell types. Visual analysis of large collections of chromatin profiles across diverse cell types, integrated with computational methods, can reveal meaningful biological insights. We developed Cistrome Explorer, a web-based interactive visual analytics tool for exploring thousands of chromatin profiles in diverse cell types. Integrated with the Cistrome Data Browser database which contains thousands of ChIP-seq, DNase-seq and ATAC-seq samples, Cistrome Explorer enables the discovery of patterns of CREs across cell types and the identification of transcription factor binding underlying these patterns.

**Availability and implementation:**

Cistrome Explorer and its source code are available at http://cisvis.gehlenborglab.org/ and released under the MIT License. Documentation can be accessed via http://cisvis.gehlenborglab.org/docs/.

**Supplementary information:**

Supplementary data are available at *Bioinformatics* online.

## 1 Introduction

Chromatin profiling genomics technologies, such as ChIP-seq, DNase-seq and ATAC-seq, have been used to reveal the genomic locations and cell type specificities of cis-regulatory elements (CREs). However, the activities of CREs are still poorly understood due to their complex mechanisms that often differ across cell types. Visual analytics of large collections of chromatin profiles, integrated with computational methods, can reveal patterns suggestive of cis-regulatory mechanisms. Existing genomics visualization tools are limited in their display of chromatin profiles. Common genome browsers (Chelaru *et al.*, 2014; Kent *et al.*, 2002; Robinson *et al.*, 2011; Skinner *et al.*, 2009; Zhou *et al.*, 2013) can only visualize tens of samples at a time, and an integrative visual analysis with sample metadata (e.g. cell types) is limited or even impossible. UCSC Xena (Goldman *et al.*, 2017) only focuses on cancer data (e.g. The Cancer Genome Atlas (TCGA) and Genomic Data Commons (GDC)). Moreover, there is no integrative visualization platform that allows users to identify genomic regions of interest to identify visual patterns across thousands of profiles, and subsequently query epigenomic databases to obtain more information about these patterns (e.g. to identify potential transcription factors).

## 2 Materials and methods

**Fig. 1.**
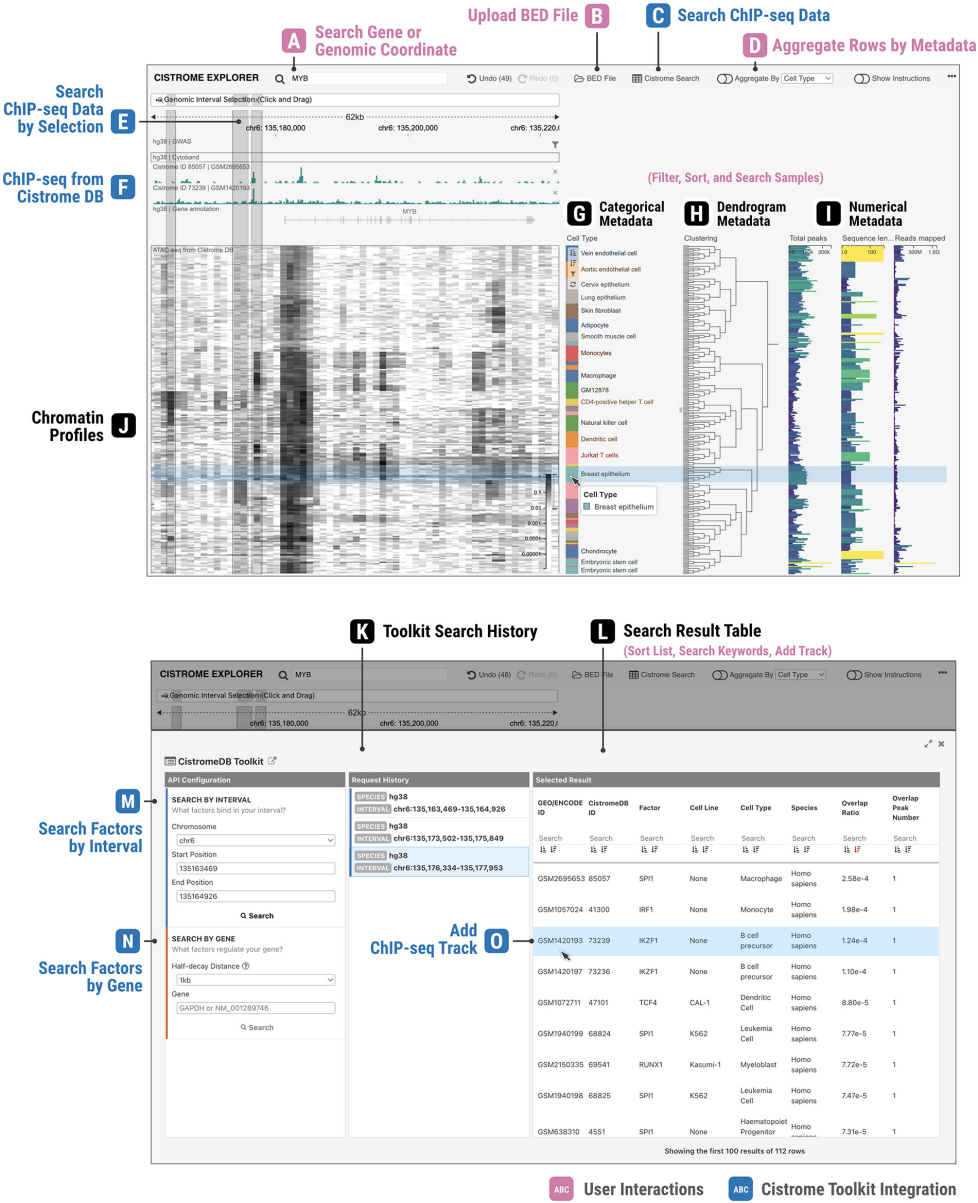
Cistrome Explorer interface. (Top) ATAC-seq profiles representing diverse cell types, 228 samples from the Cistrome DB. (Bottom) Cistrome DB Toolkit analysis, showing a list of transcription factors associated with regions that were interactively selected from the heatmap

We developed Cistrome Explorer (Fig. 1), an interactive scalable visual analytics tool for chromatin profiling data, which facilitates the exploration of chromatin accessibility, histone modifications and transcription factor binding across thousands of profiles in diverse cell types. To enable efficient analysis, Cistrome Explorer provides preprocessed chromatin profiles that are assembled from Cistrome Data Browser (Cistrome DB) (Zheng *et al.*, 2019). Cistrome Explorer can also be run locally, allowing users to visualize their own data (Supplementary Note).

**Visualizations:** Cistrome Explorer can display thousands of chromatin profiles as heatmaps. Additional tracks can be stacked on top of the heatmap to assist in the interpretation of these profiles. For example, along with gene annotations, a GWAS Catalog (Welter *et al.*, 2014) track can be included to show reported variants and linked diseases. Local bed files can be visualized using lollipop plots. By editing configuration files, users can also add other types of tracks supported by HiGlass (Kerpedjiev *et al.*, 2018) or Gosling (L’Yi *et al.*, 2021). Adjacent to the gene regulation heatmaps, corresponding metadata of individual samples can be visualized as bar charts and dendrograms that represent cell type, tissue type, quality scores and hierarchical clustering results.

**User interactions:** Cistrome Explorer supports several interactive features for effective analysis. Users can quickly change the focus of analysis in the genome view through seamless zooming and panning with the mouse or trackpad or by navigating to known genes through gene symbol lookup. For scalable analysis, Cistrome Explorer offers diverse ways to aggregate, filter and rearrange the large collection of samples. For example, users can filter out samples with low-quality scores and rearrange the remaining samples by cell types to find patterns that are cell type-specific. Users can also aggregate samples per cell type, displaying average values of individual cell types. If users find a certain sample interesting in the heatmap, it can be added as a separate bar chart track for a detailed visual inspection.

**Cistrome DB Toolkit:** To enable efficient identification of potential CREs, we integrated Cistrome DB Toolkit (Zheng *et al.*, 2019) into Cistrome Explorer. Users can access the toolkit features to search for transcription factors that are most likely to bind in a genomic region of interest or near a gene of interest. Any ChIiP-seq samples from the toolkit results can be added to the visualization as bar chart tracks for further visual inspection.

## 3 Use case

To illustrate the functionality of Cistrome Explorer, we demonstrate use cases with ATAC-seq data from Cistrome DB (Fig. 1). MYB is a transcription factor that is important in hematopoietic development (Novershtern *et al.*, 2011) and has been found to play a role in estrogen receptor-positive breast cancer (Drabsch *et al.*, 2007). After navigating to MYB, Cistrome Explorer reveals that although MYB is a transcription factor important in development, its promoter is accessible in most cell types. In the immune cell types and in breast cancer, the pattern of the enhancer near the MYB transcription start site (TSS) is complex. Using the Cistrome toolkit enables us to find potential transcription factors. One enhancer 4 kb from the TSS, accessible in monocytes, macrophages and dendritic cells, is bound by the hematopoietic regulators SPI1 and IKZF1, while another 17 kb from the TSS is bound by RELA in T cells and B-cell derived lymphoblastoid cell lines. The breast cancer-associated enhancer 6 kb from the TSS is bound by ESR1, consistent with the observation that MYB is regulated by the Estrogen Receptor (Drabsch *et al.*, 2007). Additional use cases are illustrated in Supplementary Note.

## 4 Conclusion

Cistrome Explorer is a scalable interactive visual analytics tool for gene regulation data. Visualizations for chromatic profiles and their metadata in Cistrome Explorer scale to thousands of samples. Combined with highly interactive features and integration with Cistrome DB Toolkit, users can effectively analyze the effects of CREs across diverse cell types and cell states.

## Supplementary Material

btad018_Supplementary_Data

## Data Availability

The data underlying this article are available in the article and in its online supplementary material.
