# Supplementary material for: Cistrome Explorer: an interactive visual analysis tool for large-scale epigenomic data
Source: Bioinformatics. 2023 Jan 23;39(2):btad018. doi: 10.1093/bioinformatics/btad018 (PMC9900209; doi:10.1093/bioinformatics/btad018)
Supplement: btad018_Supplementary_Data [file btad018_supplementary_data.zip › Supplementary Note.pdf]

# Supplementary Note

## List of Contents

1. Use Cases
2. Software Architecture
3. Deploying Cistrome Explorer with Your Own Data
4. Key Scoring Metrics
5. Supplementary References

## 1. Use Cases

To illustrate the utility of Cistrome Explorer, we describe multiple use cases.

### GREB1

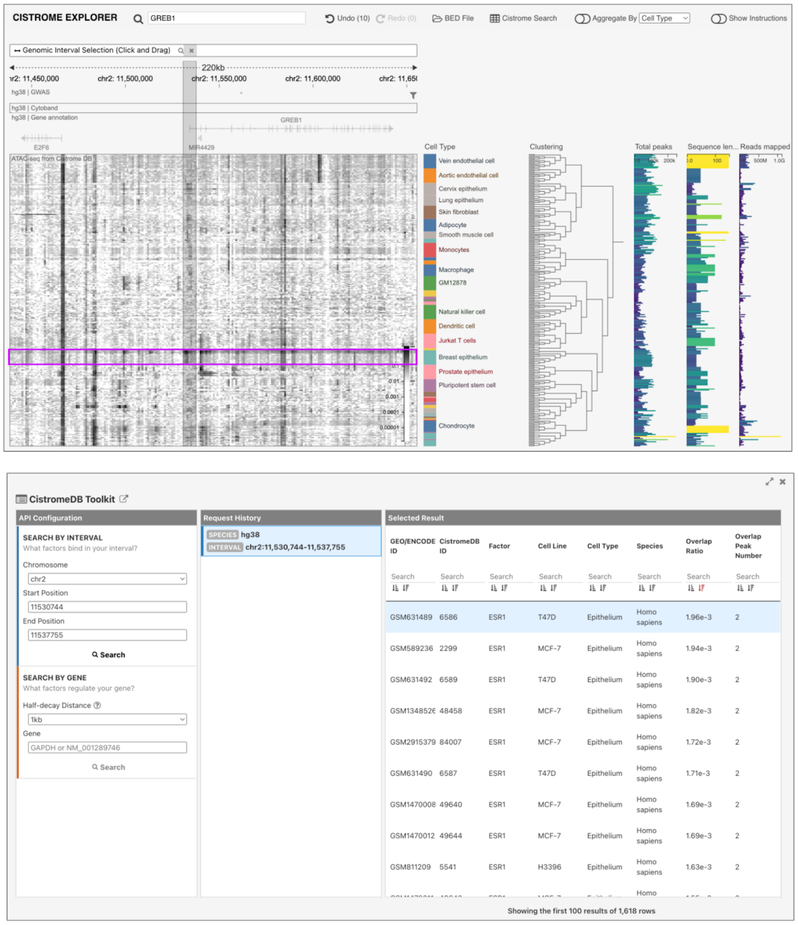


**Supplementary Fig 1.** *Top:* Cistrome Explorer Genome View showing the region around GREB1. The selected region (gray highlighting) corresponds to the promoter of GREB1.

Highlighting (purple box) shows high accessibility (darker gray in heatmap) in breast cancer cell lines. *Bottom:* Results of query against CistromeDB Toolkit shows top hits that bind in the selected promoter region (gray highlighting).

An investigator interested in the regulation of the GREB1 gene, looking at Cistrome DB ATAC-seq data in Cistrome Explorer (Supplementary Fig. 1), would immediately see that the promoter of this gene is in a closed chromatin conformation in most cell types, and has the highest level of accessibility in breast cancer cell lines. In contrast, the neighboring gene, E2F6 has a high level of promoter accessibility across all cell types. Promoter accessibility therefore plays a role in the regulation of the GREB1 gene. The user may then be interested in identifying transcription factors that regulate accessibility of the GREB1 promoter. Selecting the promoter in the Cistrome Explorer genome view and submitting the query to the Cistrome Toolkit, one can see that the Estrogen Receptor (ESR1) has been observed to bind to this region in many ChIP-seq experiments. Besides ESR1, other transcription factors binding in this region include, the Androgen Receptor (AR), the Progesterone Receptor (PR), and an additional nuclear receptor NR5A2.

## FOXA1

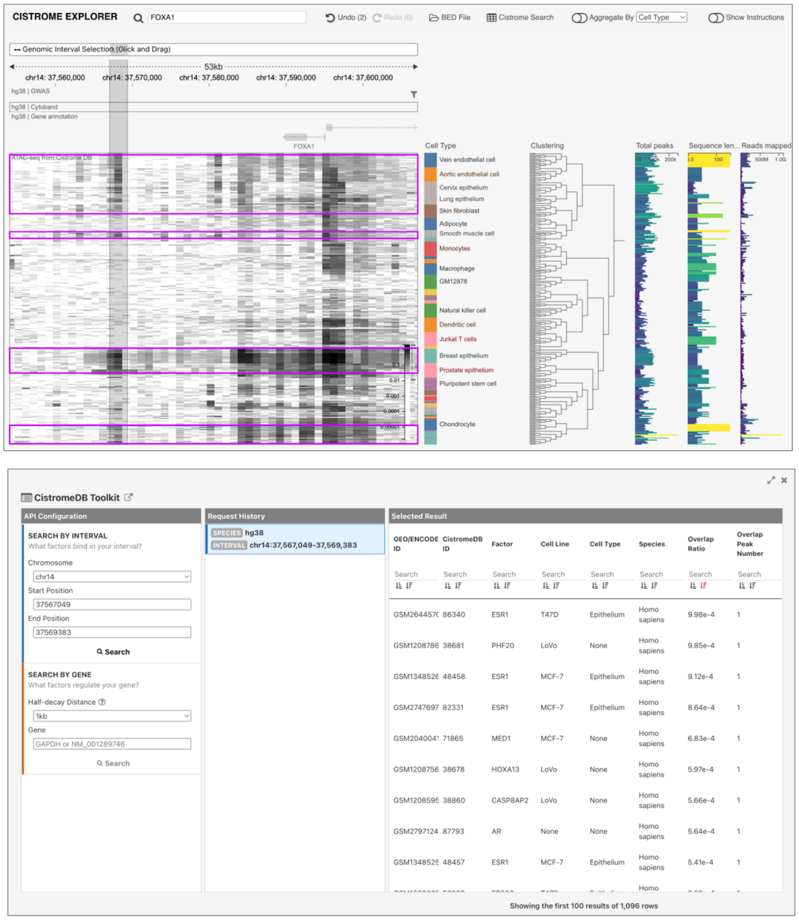


**Supplementary Fig 2.** *Top:* Cistrome Explorer Genome View showing the region around FOXA1. The selected region (gray highlighting) corresponds to the promoter of FOXA1.

Highlighting (purple box) shows cell type restricted chromatin accessibility in several regions across the locus. *Bottom:* Results of query against CistromeDB Toolkit shows top hits that bind in the selected promoter region (gray highlighting).

FOXA1 is a pioneer transcription factor that has an important role in shaping the accessible chromatin regions in estrogen receptor positive breast cancers [1]. Using the Cistrome Explorer to inspect the chromatin accessibility landscape surrounding the FOXA1 gene itself (Supplementary Fig. 2), we see that the promoter is accessible in many but not all cell types. Breast cancers show a striking pattern of cell type restricted chromatin accessibility in several regions across the locus. The Cistrome Toolkit analysis of the region chr14:37,567,049–37,569,383 reveals ESR1 and FOXA1 binding in estrogen receptor positive breast cancers. FOXA1 therefore appears to play an autoregulatory role.

**IL-6**

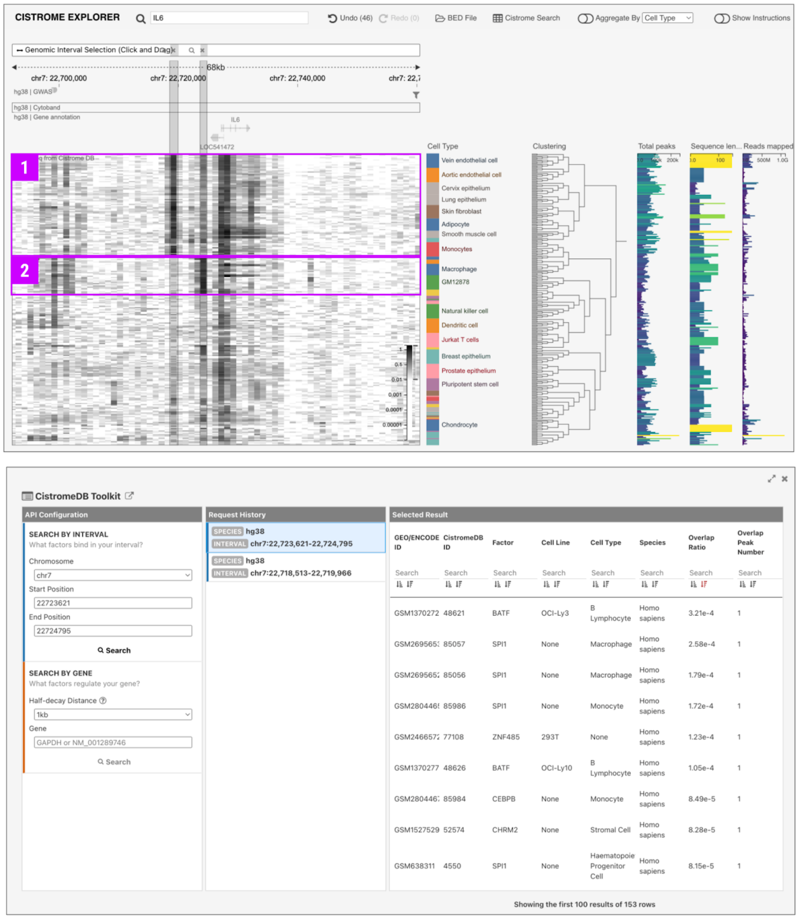


**Supplementary Fig 3.** *Top:* Cistrome Explorer Genome View showing the region around IL-6. The selected regions (gray highlighting) correspond to cis-regulatory regions outside of the promoter. Highlighting shows open chromatin (darker gray in heatmap) in vein endothelia, smooth muscle, adipocytes, and lung epithelia (purple box 1) and monocytes, macrophages, microglia, and dendritic cells (purple box 2). *Bottom:* Results of queries against CistromeDB Toolkit shows top hits that bind in the selected cis-regulatory regions (gray highlighting).

The IL-6 gene encodes a proinflammatory cytokine with key roles in immune function [2]. From the Cistrome Explorer view (Supplementary Fig. 3), the promoter of this gene is accessible in most cell types. Several cis-regulatory regions outside of the promoter, however, show tissue-restricted patterns. Monocytes, macrophages, microglia and dendritic cells have a common region of open chromatin 3kb upstream of the TSS. In a variety of other cell types, including vein endothelia, smooth muscle, adipocytes and lung epithelia there is another cis-regulatory region 8kb upstream of the TSS. Querying the Cistrome Data Browser data through the Cistrome Explorer genomic interval search feature, we find that the immune specific region is bound by the immune related factors SPI1, CEBPB and BATF. The other cis-regulatory region is bound by FOSL1, HES2 and GR in lung epithelium cell lines. IL-6 therefore appears to be regulated by at least two separate enhancer modules, one in myeloid cells, another in endothelial and epithelial cell lineages.

## GZMB

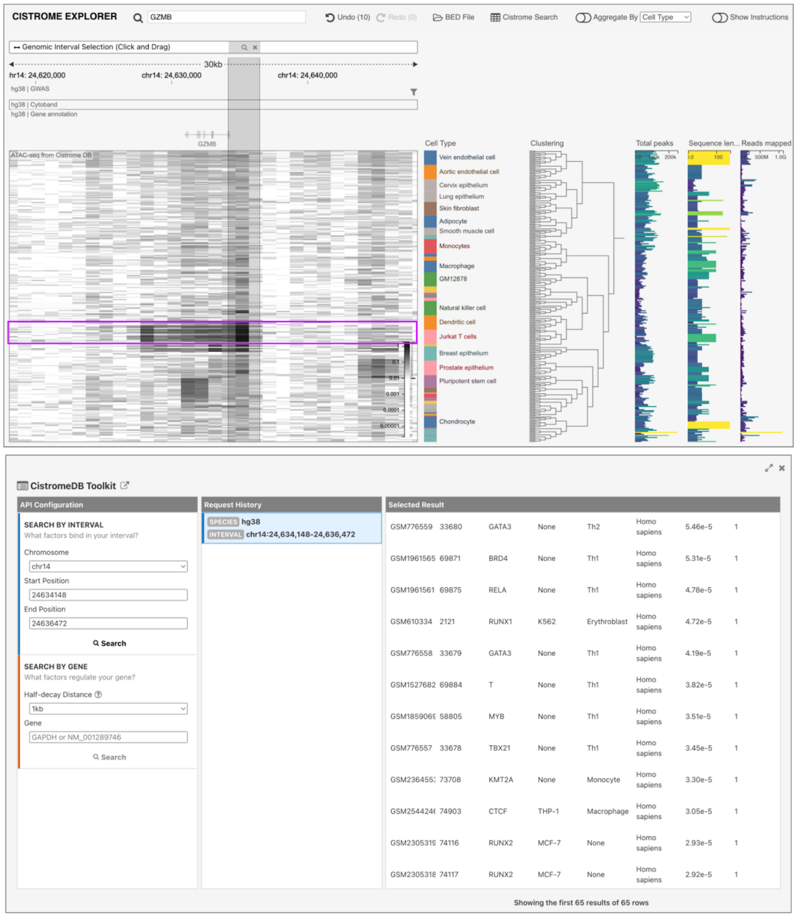


**Supplementary Fig 4.** *Top:* Cistrome Explorer Genome View showing the region around GZMB. The selected region (gray highlighting) corresponds to the promoter of GZMB. Highlighting (purple box) shows that accessible chromatin is restricted to a small number of cell types. *Bottom:* Results of query against CistromeDB Toolkit shows top hits that bind in the selected promoter region (gray highlighting).

The GZMB gene encodes a serine protease that is secreted by natural killer cells and cytotoxic T-cells to induce apoptosis in target cells [3]. The Cistrome Explorer shows (Supplementary Fig. 4) that promoter accessibility plays an important role in controlling the expression of this gene, as accessible chromatin in the promoter is restricted to a few cell types, including natural killer cells and to T-cells. The Cistrome Toolkit reveals GATA3 and MYB, TBX21, along with the cytokine responsive factors STAT5B and RELA to bind to the promoter. GATA3 is important in the development of B-cells, T-cells and natural killer cells [4], while TBX21 regulates the development of T-helper 1 cells [5].

# 2. Software Architecture

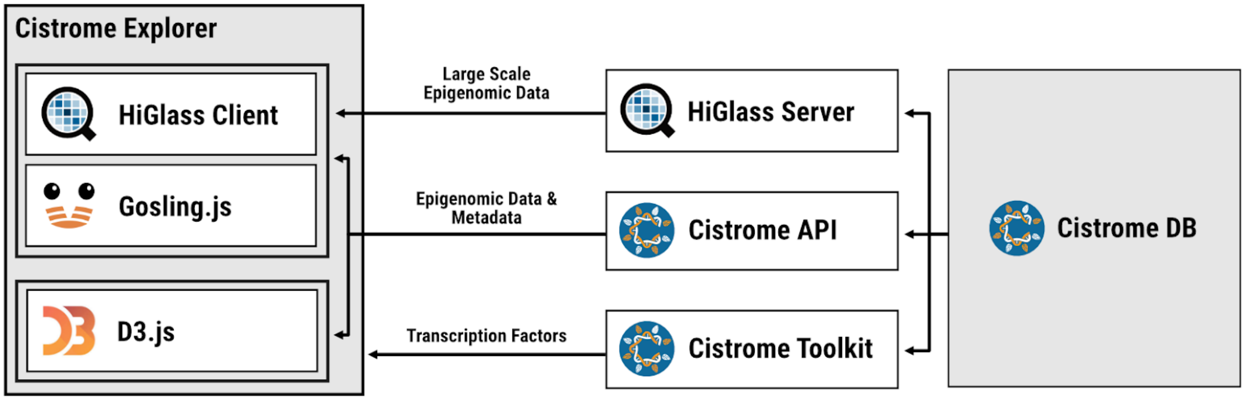


**Supplementary Fig 5.** Architecture for the Cistrome Explorer web application with connections to HiGlass and Cistrome DB.

Supplementary Fig 5. illustrates the software architecture of Cistrome Explorer. The HiGlass Client [6] and Gosling.js [7] render epigenomic data, and D3.js [8] displays metadata. Cistrome Explorer uses a large collection of epigenomic datasets from Cistrome Data Browser [9]. The preprocessed epigenomic data (i.., demo datasets) is stored in a HiGlass server[1], while additional samples and their metadata can be accessed on the fly through Cistrome APIs. Cistrome Toolkit [10] enables searching for transcription factors that potentially regulate genes of interest or bind in genomic regions of interest.

# 3. Deploying Cistrome Explorer with Your Own Data

Users can run Cistrome Explorer locally to display their own data. This can be done by following two main steps: (1) data preparation and (2) running the application locally.

## Data Preparation

Users need to prepare the epigenomic data and metadata in Cistrome Explorer compatible formats.

[1] https://docs.higlass.io/higlass_server.html

### Epigenomic Data (Multivec)

The epigenomic data is stored in the multivec format introduced by HiGlass (https://docs.higlass.io/data_preparation.html#multivec-files). This data file can be generated using the Python package clodius[2]. The preprocessing steps for multivec files are illustrated in detail in the HiGlass documentation (https://docs.higlass.io/data_preparation.html#multivec-files).

### Metadata (JSON)

Corresponding metadata are stored in a JSON format where each element of the JSON array represents metadata of a sample. Example datasets can be found in the GitHub repository[3].

### Setting Up HiGlass Server

The data displayed on the Cistrome Explorer is provided by a HiGlass server[4] which is used to store and efficiently extract requested portions of the multivec data. This server can be setup locally, and the multivec files ingested into the local server following steps that are illustrated in the documentation:

- https://docs.higlass.io/higlass_server.html#development
- https://docs.higlass.io/higlass_server.html

## Running Application Locally

To run the Cistrome Explorer client locally, you need to download the Cistrome Explorer (https://github.com/hms-dbmi/cistrome-explorer) source code. Once the dependencies have been installed, the application can be run in a web browser, following the steps explained in the README.md file (https://github.com/hms-dbmi/cistrome-explorer#development).

To use local data files that have been prepared through the Data Preparation step, two configuration files are needed:

- Visualization configuration file (https://github.com/hms-dbmi/cistrome-explorer/blob/master/src/demo/demo.js)
    - This file customizes visualizations in Cistrome Explorer, including the tracks to include in the epigenomic data visualization (e.g., Heatmap, bar charts, or any other HiGlass and Gosling tracks) and metadata visualization (e.g., what metadata to show and what visualization types to use).
    - To use an epigenomic dataset that is contained in the local HiGlass server, edit the viewConfig file.
- Metadata configuration file (https://github.com/hms-dbmi/cistrome-explorer/blob/master/src/demo/fakedata/index.js)

---

[2] https://github.com/higlass/clodius
[3] https://github.com/hms-dbmi/cistrome-explorer/tree/master/src/demo/fakedata
[4] https://docs.higlass.io/higlass_server.html

- A metadata JSON file describing the tracks in the multivec file can be specified in the visualization configuration file.
- Alternatively, the JSON metadata file can be opened directly from the application by clicking on a button named "Open Local Metadata (JSON)", which is visible after clicking on the menu button on the left-top corner of the Cistrome Explorer (Supplementary Fig. 6).

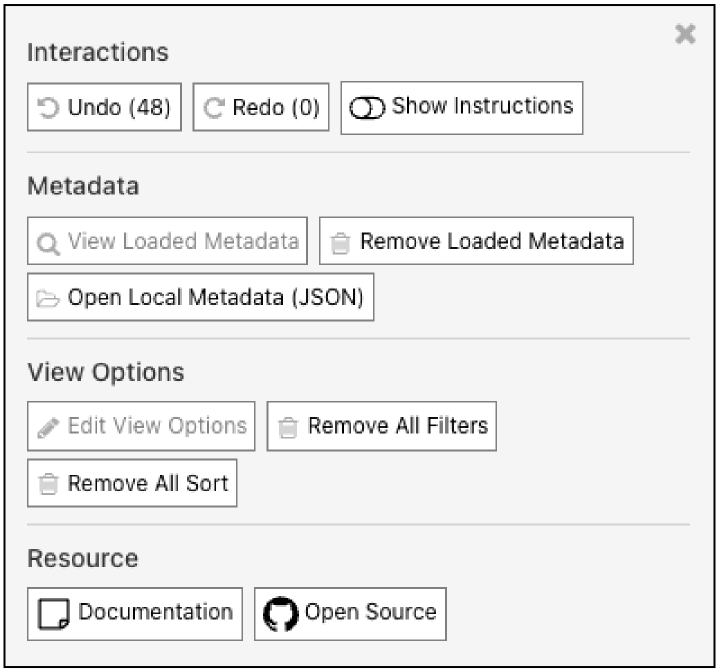


**Supplementary Fig 6.** The menu panel provides several interactions and external resources, as well as a way to load a JSON metadata file to display it in the Cistrome Explorer.

# 4. Key Scoring Metrics

This section describes scoring metrics used in the Cistrome Explorer. Full details can be found in the original papers [9,11].

## Regulatory Potential

The regulatory potential quantifies the potential that a given gene is regulated by a given transcription factor. It is calculated as a weighted sum of peaks located close to the gene transcription start site (TSS), where the weights represent the estimated influence of the peak on the gene and are determined by a function of the distance between the peak and the TSS [9,11].

## Overlap Ratio

The overlapped peak ratio is defined as an overlapped peak number divided by total peak number in the sample [10].

# 5. Supplementary References


1. Lupien M, Eeckhoute J, Meyer CA, Wang Q, Zhang Y, Li W, et al. FoxA1 translates epigenetic signatures into enhancer-driven lineage-specific transcription. Cell. 2008;132: 958–970.
2. Jones SA, Jenkins BJ. Recent insights into targeting the IL-6 cytokine family in inflammatory diseases and cancer. Nat Rev Immunol. 2018;18: 773–789.
3. Lord SJ, Rajotte RV, Korbutt GS, Bleackley RC. Granzyme B: a natural born killer. Immunol Rev. 2003;193: 31–38.
4. Wan YY. GATA3: a master of many trades in immune regulation. Trends Immunol. 2014;35: 233–242.
5. Lazarevic V, Glimcher LH, Lord GM. T-bet: a bridge between innate and adaptive immunity. Nat Rev Immunol. 2013;13: 777–789.
6. Kerpedjiev P, Abdennur N, Lekschas F, McCallum C, Dinkla K, Strobelt H, et al. HiGlass: web-based visual exploration and analysis of genome interaction maps. Genome Biol. 2018;19: 125.
7. L'Yi S, Wang Q, Lekschas F, Gehlenborg N. Gosling: A Grammar-based Toolkit for Scalable and Interactive Genomics Data Visualization. IEEE Trans Vis Comput Graph. 2021;PP. doi:10.1109/TVCG.2021.3114876
8. Bostock M, Ogievetsky V, Heer J. $D^3$ data-driven documents. IEEE transactions on. 2011. Available: https://ieeexplore.ieee.org/abstract/document/6064996/
9. Zheng R, Wan C, Mei S, Qin Q, Wu Q, Sun H, et al. Cistrome Data Browser: expanded datasets and new tools for gene regulatory analysis. Nucleic Acids Res. 2019;47: D729–D735.
10. Zheng R, Dong X, Wan C, Shi X, Zhang X, Meyer CA. Cistrome Data Browser and Toolkit: analyzing human and mouse genomic data using compendia of ChIP-seq and chromatin accessibility data. Quantitative Biology. 2020;8: 267–276.
11. Wang S, Sun H, Ma J, Zang C, Wang C, Wang J, et al. Target analysis by integration of transcriptome and ChIP-seq data with BETA. Nat Protoc. 2013;8: 2502–2515.
